# Evaluating the Safety of Potential Probiotic *Enterococcus durans* KLDS6.0930 Using Whole Genome Sequencing and Oral Toxicity Study

**DOI:** 10.3389/fmicb.2018.01943

**Published:** 2018-08-21

**Authors:** Bailiang Li, Meng Zhan, Smith E. Evivie, Da Jin, Li Zhao, Sathi Chowdhury, Shuvan K. Sarker, Guicheng Huo, Fei Liu

**Affiliations:** ^1^Key Laboratory of Dairy Science, Ministry of Education, Northeast Agricultural University, Harbin, China; ^2^Food Science and Nutrition Unit, Department of Animal Science, Faculty of Agriculture, University of Benin, Benin City, Nigeria; ^3^Department of Food Science, Food College, Northeast Agricultural University, Harbin, China

**Keywords:** probiotic, *Enterococcus durans*, safety evaluation, genome, oral toxicity study

## Abstract

*Enterococcus durans* KLDS6.0930 has previously been shown to have probiotic potential. However, being a potential clinical pathogen, it becomes necessary to evaluate its safety status for novel potential probiotic use. The purpose of this study is to systematically evaluate the safety of *E. durans* KLDS6.0930 based on its genomics, phenotypic characteristics and oral toxicity. The complete genome of *E. durans* KLDS6.0930 was sequenced and analyzed for safety-related genes. Antibiotic susceptibility and the production of harmful metabolites were tested. A 28-day repeated oral dose toxicity test was implemented in rats. *In vitro, E. durans* KLDS6.0930 was resistant to five antibiotics, with intrinsic resistances to four antibiotics and no identified genes for the last. *E. durans* KLDS6.0930 was not hemolytic and virulence factors were non-functional in its genome. *E. durans* KLDS6.0930 produced a small amount of tyramine and phenethylamine; genes encoding tyramine decarboxylase were identified. In addition, genotype and phenotype analyses showed that the strain did not have the ability to generate D-lactic acid, indole, or nitroreductase. *In vivo, E. durans* KLDS6.0930 did not induce adverse effects on the organs, hematological and serum biochemical parameters, or cecal bacterial populations in the oral toxicity test. These results indicate that *E. durans* KLDS6.0930 can be safely used as a potential probiotic for human consumption and animal feed.

## Introduction

*Enterococcus*, a large genus of lactic acid bacteria (LAB), includes 54 species characterized as gram-positive, facultative anaerobic, and non-spore forming (Foulquié Moreno et al., 2006; Van Tyne and Gilmore, 2014). Enterococci are long-standing and non-pathogenic commensal bacteria in the gastrointestinal tract (GIT) of humans and animals (Byappanahalli et al., 2012; Weng et al., 2013; Jahan et al., 2015). They are among the first LAB to colonize the neonatal GIT (Fanaro et al., 2003) and could be involved in the development of the human microbiome (Dominguez-Bello et al., 2010). Enterococci are also widely distributed in foods and the environment, playing important roles in shelf-life improvement and the development of both flavor and texture when present in fermentation foods such as certain cheeses and fermented sausages (Rea et al., 2004; Gaglio et al., 2016; Santos et al., 2017). Furthermore, some *Enterococcus* spp. have been shown beneficial properties, such as immune system regulation, normal intestinal microflora maintenance, antitumor activity, antimicrobial activity, antioxidant activity, and lowering cholesterol levels (Pieniz et al., 2014; Molina et al., 2015; Guo et al., 2016; Li P. et al., 2017). They are therefore frequently used as probiotics to promote human and animal health, or treat diseases such as diarrhea, irritable bowel syndrome, or antibiotic-associated diarrhea (Franz et al., 2011).

Despite these benefits, some enterococci are also considered as opportunistic zoonotic pathogens, associated with nosocomial infections like urinary tract infections (the second most common causative agent), bacteraemia (the third most commonly isolated agent), surgical site infections, bloodstream infections, endocarditis and diarrhea (Schaberg et al., 1991; Foulquié Moreno et al., 2006). In addition, production of hazardous compounds by some enterococcal strains can be toxic in food production (Franz et al., 2001; Choi and Woo, 2013; Camargo et al., 2014). Due to properties of enterococci as both beneficial organisms and nosocomial pathogens, verifying the safety of a novel probiotic strain is of utmost importance. However, simple safety evaluations of enterococci are difficult because they usually carry multiple antibiotic resistances and virulence factors (EFSA, 2008). For this reason, comprehensive safety assessment of *Enterococcus* must be taken into consideration.

*E. durans* KLDS6.0930 strain evaluated in this study was originally isolated from traditional naturally fermented cream from Inner Mongolia in China. Previous *in vitro* and *in vivo* studies indicated that *E. durans* KLDS6.0930 possesses potential probiotic properties included acid and bile tolerance, adherence to Caco-2 cells and a cholesterol-lowering effect (Guo et al., 2016). The genome of *E. durans* KLDS6.0930 was the first whole genome sequence of *E. durans* and has been used as representative genome by GenBank. Genetic elements with potential probiotic properties were exploited in our previous report (Liu et al., 2016). However, to guarantee the safe use of this strain as a potential probiotic or bioprotective adjunct culture, it is necessary to comprehensively evaluate any potential adverse effects. Thus, the aim of this study is to fully assess the safety status of *E. durans* KLDS6.0930 using genomic data, phenotypic assays and oral toxicity studies.

## Materials and methods

### Bacterial strains and culture conditions

*E. durans* KLDS6.0930 was isolated from traditional naturally fermented cream from Inner Mongolia in China and was made available at the Key Laboratory of Dairy Science (KLDS), Northeast Agricultural University (NEAU), Harbin, China. *E. durans* KLDS6.0930 was maintained in M17 broth (Oxoid Ltd, Basingstoke, UK) with 10% glycerol and stored at −20°C. Before use, *E. durans* KLDS6.0930 was activated through three propagation steps in M17 broth at 37°C for 24 h. Bacteria were harvested by centrifugation at 5,000 g for 10 min. Cell pellets were then washed twice with sterile normal saline and resuspended at the desired concentration in sterile normal saline. *Escherichia coli* ATCC 25922 and *Staphylococcu*s *aureus* ATCC 25923 were incubated in Luria-Bertani (LB) broth and Tryptic Soy Broth (TSB) under aerobic conditions at 37°C, respectively.

### Genome sequencing and taxonomy

*E. durans* KLDS6.0930 genomic DNA was extracted using the DNeasy Tissue kit (Qiagen, Hilden, Germany) following the manufacturer's instruction. Detailed information on the genome sequencing study can be found in our previous report (Liu et al., 2016). The 16S rRNA gene sequence was compared with closely related sequences from GenBank using BLASTN and aligned using CLUSTALW software (Thompson et al., 1997). The phylogenetic tree was reconstructed using the neighbor-joining method with bootstrap analysis based on 1,000 resamplings using MEGA 7.0 package software (Kumar et al., 2016). The average nucleotide identity (ANI) of the two genomic sequences was evaluated by ANI calculator using the OrthoANIu algorithm (Yoon et al., 2017). The availability of the whole genome of *E. durans* KLDS6.0930 allowed the development of the ribosomal multilocus sequence typing (rMLST) scheme, which was based on 49 genes encoding the bacterial ribosome protein subunits (*rps* genes), as performed in rMLST database website (http://pubmlst.org/rmlst/). The ribosomal protein sequences retrieved from the genomes were aligned with CLUSTALW software, while neighbor-joining method was used to infer the phylogenetic tree.

### Genomic analysis of safety-related genes

The predicted genes of *E. durans* KLDS6.0930 were compared with the comprehensive antibiotic resistance database (CARD, http://arpcard.mcmaster.ca/) (Mcarthur et al., 2013) and virulence factors database (VFDB, http://www.mgc.ac.cn/VFs/main.htm) (Chen et al., 2016) for identifying antibiotic resistance and virulence factor. Furthermore, the ResFinder 3.0 (https://cge.cbs.dtu.dk//services/ResFinder/) (Zankari et al., 2012) and PathogenFinder 1.1 (https://cge.cbs.dtu.dk/services/PathogenFinder/) (Cosentino et al., 2013) were used for identifying the acquired antibiotic resistance genes and pathogenicity factors, respectively. Clustered regularly interspersed short palindromic repeats (CRISPR) and prophage sequences were identified by CRISPR Finder (http://crispr.i2bc.paris-saclay.fr/Server/) (Grissa et al., 2007) and PHASTER (http://phaster.ca/) (Arndt et al., 2016), respectively. The predicted genes were annotated against the restriction enzyme database (REBASE, http://tools.neb.com/genomes/) (Roberts et al., 2015) for searching restriction enzyme. The gene sequences associated to the adverse metabolites such as amino acid decarboxylases, nitroreductase, and D-lactate dehydrogenase, were referenced from Genbank and inquired the genome by BLASTN.

### Antibiotic susceptibility assay

Minimum inhibitory concentrations (MICs) for antibiotics (ampicillin, penicillin, vancomycin, erythromycin, tetracycline, gentamicin, kanamycin, clindamycin, ciprofloxacin, trimethoprim, and rifampicin) against *E. durans* KLDS6.0930 were determined using a 96-well plate gradient dilution method (Guo et al., 2016a). Susceptibility was determined on the criteria adopted by European Food Safety Authority (EFSA) for the assessment of bacterial resistance to antibiotics (EFSA, 2012a).

### Hemolytic activity

The hemolytic activity of *E. durans* KLDS6.0930 was tested by incubating bacteria on blood agar (7% v/v sheep blood) for 48 h at 37°C (Pieniz et al., 2014). α-hemolysis (green-hued halo around the colonies) or β-hemolysis(clear halo around colonies) implied positive hemolytic activity. γ-hemolysis (absence of clearing zone surrounding colonies) was classified as a negative result. *S. aureus* ATCC 25923 was used as a positive control.

### Biofilm formation

Biofilm formation was evaluated using a previously described method (Kopit et al., 2014) with some modifications. Briefly, bacteria was grown overnight in M17 broth at 37°C. After 24 h, 180 μL fresh M17 broth and 20 μL cell suspension were combined in a sterile 96-well polystyrene microtiter plate. A negative control using 200 μL M17 broth alone was also added. *S. aureus* ATCC 25923 was used as a positive control. After incubation of the strain at 37°C for 24 h, wells were washed two times with sterile saline, inverted for 15 min to dry and stained with 1% (w/v) crystal violet for 15 min. The wells were then rinsed again with sterile saline and filled with 200 μL of acetone and ethanol solution (20:80, v/v). Absorbance at 595 nm was measured by using a microplate reader (Model 680 BIO-RAD, China). The cut-off OD (ODc) was defined as three standard deviations above the mean OD of the negative control. The ability to form biofilm was classified according to Stepanovic et al. (2000) as follows: OD ≤ ODc as non-biofilm-producer (0), ODc < OD ≤ 2 OD as weak biofilm producer (+), 2 ODc < OD ≤ 4 ODc as moderate biofilm producer (++), and OD ≥4 ODc as strong biofilm producer (+++).

### Detection of harmful metabolites

Biogenic amines produced by *E. durans* KLDS6.0930 were quantified by high performance liquid chromatography (HPLC). Pretreatment and dansyl chloride derivatization of samples were performed as previously reported (Dadáková et al., 2009; Lorencová et al., 2012). HPLC measurements were performed on Waters Alliance HPLC system (Waters e2695, USA) equipped with a binary pump and a UV/Vis detector (λ = 245 nm). Samples were separated on a C-18 column (250 mm × 4.6 mm, 5 μm, Japan). Separation and examination of biogenic amines were performed according to Smělá et al. (2004). Chromatograms of biogenic amines (phenylethylamine, tryptamine, cadaverine, putrescine, histamine, tyramine, spermine, and spermidine) in standard solution are presented in Figure S1.

The optical purity of the resulting lactic acid was estimated using an L/D-lactic acid enzymatic test kit following the manufacturer's instructions (Megazyme, Ireland). The ability of *E. durans* KLDS6.0930 to produce indole was evaluated using Kovacs's indole kit (Hopebio-Technology Co., Ltd, China). A red ring was regarded as positive. *E. coli* ATCC25922 was used as a positive control. The activity of nitroreductase was determined using the assay kit according to the manufacturer's instructions (Nanjing Jiancheng Bioengineering Institute, China). *E. coli* ATCC25922 was used as a positive control.

### Animals

Specific Pathogen Free (SPF) Sprague-Dawley (SD) rats aged 6–7 weeks were supplied by Vital River Laboratory Animal Technology Co., Ltd. (Beijing, China). All rats were randomly put into plastic cages in an animal chamber under controlled environmental conditions with a 12-h light/dark cycle at a temperature of 23 ± 2°C and a relative humidity of 50 ± 20%. The rats were acclimatized to the laboratory conditions for 1 week before the start of the experiment and permitted with free access to drinking water and standard diet. The experimental protocol was approved by the guidelines of Animal Care and Use Committee of Northeast Agricultural University (SRM-06).

### Subacute oral toxicity

This study was performed according to Organization for Economic Co-operation and Development (OECD) test guidelines (OECD, 2008). Male and female rats were randomly divided into two groups of six rats for each gender. Rats in the treatment group were orally gavaged with 1 mL *E. durans* KLDS6.0930 at 1 × 10^9^ CFU/kg body weight (BW) once daily for 28 days. The control group was administered with 1 mL sterile normal saline. During the treatment period, all rats were observed daily for clinical signs of morbidity and mortality. BW and feed intake were monitored weekly. At the end of the dosing period, all rats were deprived of food and water for 16 h before humane sacrifice under diethyl ether anesthesia.

### Hematological and serum biochemistry analyses

Hematological variables were analyzed in all rats at the end of the treatment period. Blood samples were collected in heparinized tubes to assess red blood cell count (RBC), white blood cell count (WBC), hemoglobin (HGB), platelet count (PLT), mean corpuscular volume (MCV), mean corpuscular hemoglobin concentration (MCHC), neutrophils, monocytes, lymphocytes, and eosinophils using an automatic hematological analyzer (Nihon Kohden, Japan).

Whole blood was centrifuged to obtain serum for analysis on an automatic biochemistry analyzer (Toshiba, Japan) for the following parameters: aspartate aminotransferase (AST), alanine aminotransferase (ALT), alkaline phosphatase (ALP), total bilirubin (TBIL), total protein (TP), albumin (ALB), glucose (GLU), triglycerides (TG), total cholesterol (TC), high density lipoprotein cholesterol (HDL), low density lipoprotein cholesterol (LDL), urea (UREA), creatinine (CRE), sodium (Na), chloride (Cl), calcium (Ca), and inorganic phosphorus (P).

### Gross necropsy and histopathological examination

Organs including heart, spleen, liver, kidney, lung, brain, adrenal thymus, epididymides (male rats), testes (male rats), ovaries (female rats), and uterus (female rats) were collected for gross necropsy and weighed. The organ index was calculated as a percentage of organ weight to terminal BW. Selected organs (heart, spleen, liver, kidney, lung, brain, and jejunum) were excised and fixed in 10% neutral buffered formalin for histopathology. The samples were then embedded in paraffin, cut into 5 μm thickness slices, and stained with hematoxylin-eosin for microscopic examination.

### Cecal microbiota

Total microbiota genomic DNA was extracted from cecal contents using a QIAamp DNA stool mini kit (Qiagen, Germany). Isolated DNA was examined by agarose gel electrophoresis and quantified using an ND-2000C spectrophotometer (NanoDrop, USA). Modified fusion primers 515F (5′-GTGCCAGCMGCCGCGGTAA-3′) and 806R (5′-GGACTACHVGGGTWTCTAAT-3′), containing a 6-bp error-correcting barcode were used to amplify the V4 hypervariable region of the bacterial 16S rRNA gene. The PCR products were quantified using an Agilent DNA 1000 Kit on an Agilent 2100 Bioanalyser (Agilent Technologies, USA), pooled together and sequenced using the Illumina Miseq (Illumina, USA). *In silico* analysis of sequenced data was performed using quantitative insights into microbial ecology (QIIME) (Caporaso et al., 2010).

### Cecal colonization

*E. durans* KLDS6.0930 at 2 × 10^8^ CFU/mL was fluorescently labeled with carboxyfluorescein diacetate succinimidyl ester (cFDA-SE) (Beyotime, China) according to the manufacturer's instructions. Male rats were randomly divided in two groups of six rats each. The control group received 1 mL normal saline. The experimental group was treated with 2 × 10^8^ CFU *E. durans* KLDS6.0930 labeled with cFDA-SE at by gavage in 1 mL of sterile normal saline. After administration for 1 day, test rats were anesthetized with diethyl ether and sacrificed for cecum tissue collection. Cecal contents were carefully removed, and the cecal wall was rinsed with 1 mL sterile normal saline. Samples were stored in the dark and subjected to flow cytometry (BD LSR Fortessa) using a 488 nm laser.

### Statistical analysis

All experiments were conducted at least in triplicate using independent assays. Values are expressed as means ± the standard deviation (*SD*). Statistical significance of data comparisons was calculated by an unpaired Student's *t*-test in the subacute toxicity assays. Values of *P* < 0.05 were considered to be statistically significant.

## Results

### Taxonomic identification and defense systems

**Figure 1:**
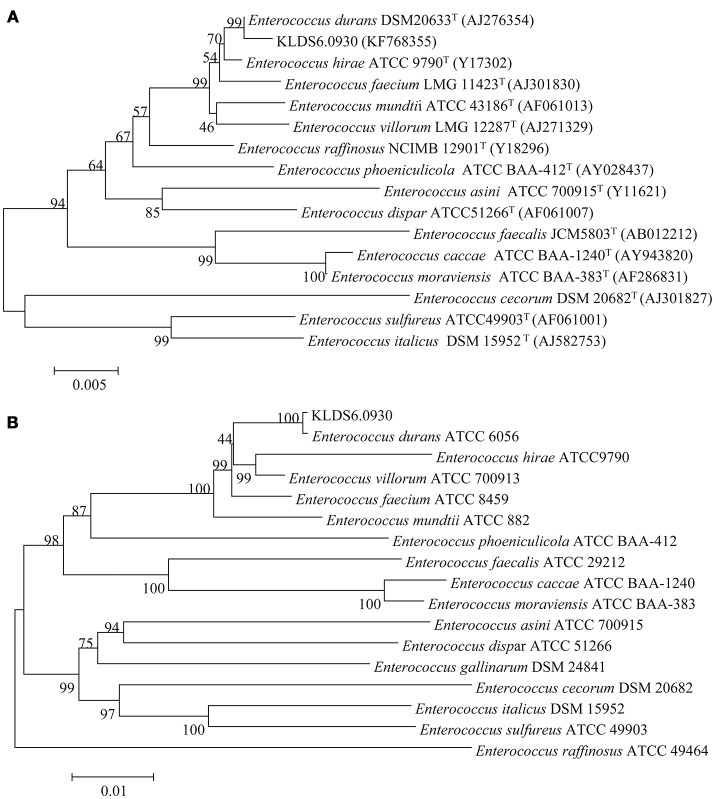
Phylogenetic trees of 16S rRNA gene **(A)** and concatenated 49 ribosomal protein sequences **(B)** using MEGA 7.0 software with the neighbor-joining method. Bootstrap values based on 1,000 resampled datasets are shown at branch nodes.

At the genomic level, the ANI value between the strain KLDS6.0930 and *E. durans* ATCC6056 was 99.62%. This agrees with the phylogenetic analysis based on 16S rRNA gene sequence (Figure 1A) and the concatenation of 49 ribosomal protein sequences (Figure 1B). These taxonomic results indicated that strain KLDS6.0930 should be assigned to species *E. durans*.

**Figure 2:**
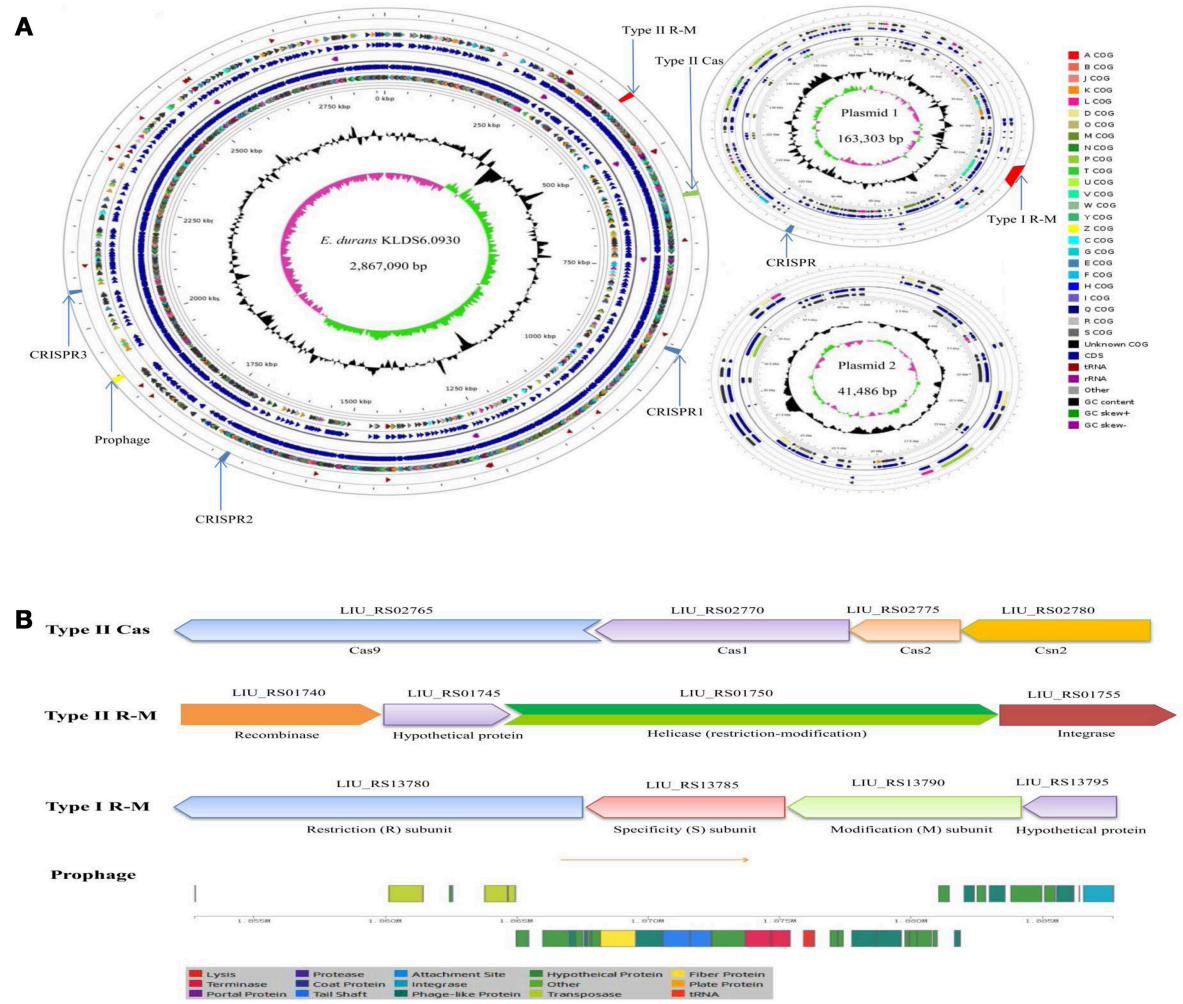
Circular genome map and defense systems of *Enterococcus durans* KLDS6.0930. **(A)** Circular genome map. From periphery to center: Defense system and prophage, tRNA, Protein coding genes (CDSs) on forward strand colored according to the assigned COG classes, Genes on forward strand, rRNA, Genes on reverse strand, CDSs on reverse strand colored according to the assigned COG classes, Genome position in kbp, GC content; GC skew (G-C)/(G + C); **(B)** Organizations of the Type II cas, Type II R-M, Type I R-M, and prophage operons.

Two circular plasmids that do not encode any genes associated with true behavioral risk factors were detected in *E. durans* KLDS6.0930 (Figure 2A). Only a prophage region (GC content, 34.84%; length, 34.9 kb) with phage attachment sites (*attL* and *attR*) in the *E. durans* KLDS6.0930 chromosome (GC content, 38.00%; length, 2.87 Mb) was identified. This region contained 36 proteins including integrase, transposase, terminase, phage-like protein, tail protein, fiber protein, lysis protein, and hypothetical protein (Figure 2B).

*In silico* analysis showed that the defense system of *E. durans* KLDS6.0930 confers immunity against mobile elements. Results obtained from the CRISPR finder database displayed that three potential CRISPR arrays are distributed on the chromosome and one confirmed CRISPR array is located on plasmid 1 (Figure 2A). Additionally, a gene cluster (*cas9, cas1, cas2, csn2*) encoding Type II-A CRISPR associated enzymes (Cas) was found in the genome of *E. durans* KLDS6.0930 (Figure 2B). Genes encoding restriction enzymes and methyltransferases in *E. durans* KLDS6.0930 were determined by comparison against the REBASE database. As shown in Figures 2A,B, one Type II restriction-modification (R-M) system and one Type I R-M were observed in the genome and plasmid 1, respectively. These data indicated that the defense systems may endow *E. durans* KLDS6.0930 with distinct genetic stability.

### Antibiotic resistance and associated genes

Antibiotic resistance-related genes in *E. durans* KLDS6.0930 were identified by searching the CARD using the perfect and strict algorithms (Table 1). The genome of *E. durans* KLDS6.0930 was found to contain one curated aminoglycoside resistance gene and 12 putative resistance genes associated with resistance to beta-lactams (4), lincosamide (2), tetracycline (2), rifampin (1), aminocoumarin (1), trimethoprim (1), and peptide (1). Regarding the possibility of acquired resistance by horizontal gene transfer (HGT), the ResFinder software did not detect any acquired antibiotic resistance genes.

**Table 1:** Putative antibiotics resistance genes in the *Enterococcus durans* KLDS6.0930 genome by searching with the CARD.

**Gene ID**	**Cut off**	**Evalue (%)**	**Identity (%)**	**ARO_name**	**ARO_category**
LIU_RS10805	Strict	0	47.87	PBP1b	Beta-lactam resistance gene
LIU_RS05210	Strict	2.24E-178	39.42	PBP2x	Beta-lactam resistance gene
LIU_RS07610	Strict	0	55.41	PBP1a	Beta-lactam resistance gene
LIU_RS05885	Strict	7.87E-159	40.69	PBP2b	Beta-lactam resistance gene
LIU_RS02535	Strict	0	54.74	lmrD	Lincosamide resistance gene
LIU_RS02530	Strict	0	55.94	lmrC	Lincosamide resistance gene
LIU_RS06275	Strict	2.41E-135	32.71	mprF	Peptide antibiotic resistance gene
LIU_RS01575	Strict	0	74.74	rpoB mutants	Rifampin resistance gene
LIU_RS06605	Strict	0	52.26	gyrB	Aminocoumarin resistance gene
LIU_RS06485	Strict	2.88E-99	87.58	dfrE	Trimethoprim resistance gene
LIU_RS12375	Perfect	1.28E-133	100	AAC(6')-Iih	Aminoglycoside resistance gene
LIU_RS08465	Strict	1.59E-61	33.94	tet42	Tetracycline resistance gene
LIU_RS06840	Strict	4.65E-96	38.95	mepA	Tetracycline resistance gene

Susceptibility of *E. durans* KLDS6.0930 to antibiotics was determined by measuring MICs, and the results were compared to the cut-off values for *Enterococcus* species as defined by EFSA (2008, 2012a). *E. durans* KLDS6.0930 was found to be resistant to gentamicin, kanamycin, clindamycin, ciprofloxacin, and rifampicin but was susceptible to ampicillin, penicillin, vancomycin, trimethoprim, and erythromycin (Table 2). The MIC of tetracycline was equal to the cut-off value.

**Table 2:** Minimum inhibitory concentrations (MICs) of *Enterococcus durans* KLDS6.0930 toward antimicrobials and the microbiological cut-off values.

**Antimicrobials**	**MIC**	**Cut-off values**
Ampicillin	< 1	4
Penicillin	< 1	4
Vancomycin	2	4
Erythromycin	2	4
Tetracycline	2	2
Gentamicin	>1024	32
Kanamycin	>1024	512
Clindamycin	>256	8
Trimethoprim	16	32
Ciprofloxacin	8	4
Rifampicin	64	32

### Putative virulence factors and assigned phenotype

Virulence genes contribute the pathogenicity to microorganisms. In order to mine possible virulence genes in the genome of *E. durans* KLDS6.0930, protein coding sequences (CDSs) were aligned to VFDB using BLASTP. The results showed that *E. durans* KLDS6.0930 genome and plasmid 1 carry a total of 45 putative virulence factors (Table S1), most of them are cell surface factors associated with host or surface adhesion and promoting biofilm formation.

The genome of *E. durans* KLDS6.0930 contains two genes encoding *efa* (LIU_RS03220 and LIU_RS03820), which contributes to adherence to extracellular matrix proteins and may cause endocarditis (Lowe et al., 1995). However, in the absence of other virulence factors, its presence poses no threat, since this gene was also found in starter *E. faecium* strains, which have a long record of safe use in food (Eaton and Gasson, 2001).

*E. durans* KLDS6.0930 harbors genes encoding two types of pili, PilA and PilB. A three-gene locus (*pilA, pilE*, and *pilF*), encoding PilA-type pilus structures was present in plasmid 1 of *E. durans* KLDS6.0930. Non-health care-associated *E. faecium* strains have been reported to express PilA, PilE, and PilF as cell wall-anchored proteins without forming pilus structures (Hendrickx et al., 2010). An operon of three genes, *ebpA, ebpB*, and *ebpC*, encoding the pilus subunits of PilB, was found in the genome of *E. durans* KLDS6.0930. However, the transcriptional regulator *ebpR* that is the promoter for the co-transcribed *ebp* operon is a pseudogene in the strain *E. durans* KLDS6.0930. Thus, pili were not detected on the surface of *E. durans* KLDS6.0930.

Biofilm formation is thought to be associated with the operon *bopABCD*, which appears to be regulated by the Fsr system through quorum sensing (Creti et al., 2006; Di Rosa et al., 2006). The operon *bopABCD* was found in the genome of *E. durans* KLDS6.0930, but *bopD* involved in transcriptional regulation of biofilm formation is a pseudogene. Moreover, *fsr* was absent in *E. durans* KLDS6.0930 genome. A biofilm formation assay showed that *E. durans* KLDS6.0930 was a weak biofilm producer, while *S. aureus* ATCC 25923 was classified as a strong biofilm producer (Table S2).

The term virulence factor refers to elements allowing a microorganism to colonize a host, contributing to the initiation and development of infection processes. Thus, it applies to secreted proteins, cell surface structures, and hydrolytic enzymes that contribute not only to bacterial pathogenicity but also to adhesion and protection (Wassenaar et al., 2015). Remarkably, many probiotic-related traits could be associated to virulence factors; therefore, *in silico* analyses must be considered in the appropriate context.

The aforementioned cell surface factors are involved in cell adhesion and intestinal colonization, which are considered potentially important for the initiation of infections in pathogens (Lowe et al., 1995; Mohamed and Huang, 2007; Sillanpää et al., 2010). As such, they are not offensive virulence factors, therefore, they could not be considered really harmful in probiotics. On the contrary, the ability to adhere to the intestinal wall is considered a desirable property for probiotics since it can allow the colonization of the GIT. Cell adhesion is a multistep process involving contact between the bacterial cell membrane and interacting surfaces. Despite the aforementioned cell surface factors, the genome of *E. durans* KLDS6.0930 also carries two fibronectin-binding proteins (LIU_RS07910 and LIU_RS10480) and an S-layer protein (LIU_RS11695), which may contribute to its adhesion. In addition, our previous study (Guo et al., 2016) showed that *E. durans* KLDS6.0930 could adhere to Caco-2 cells *in vitro*. As described below, we also demonstrated that *E. durans* KLDS6.0930 could colonize the cecum *in vivo*.

**Figure 3:**
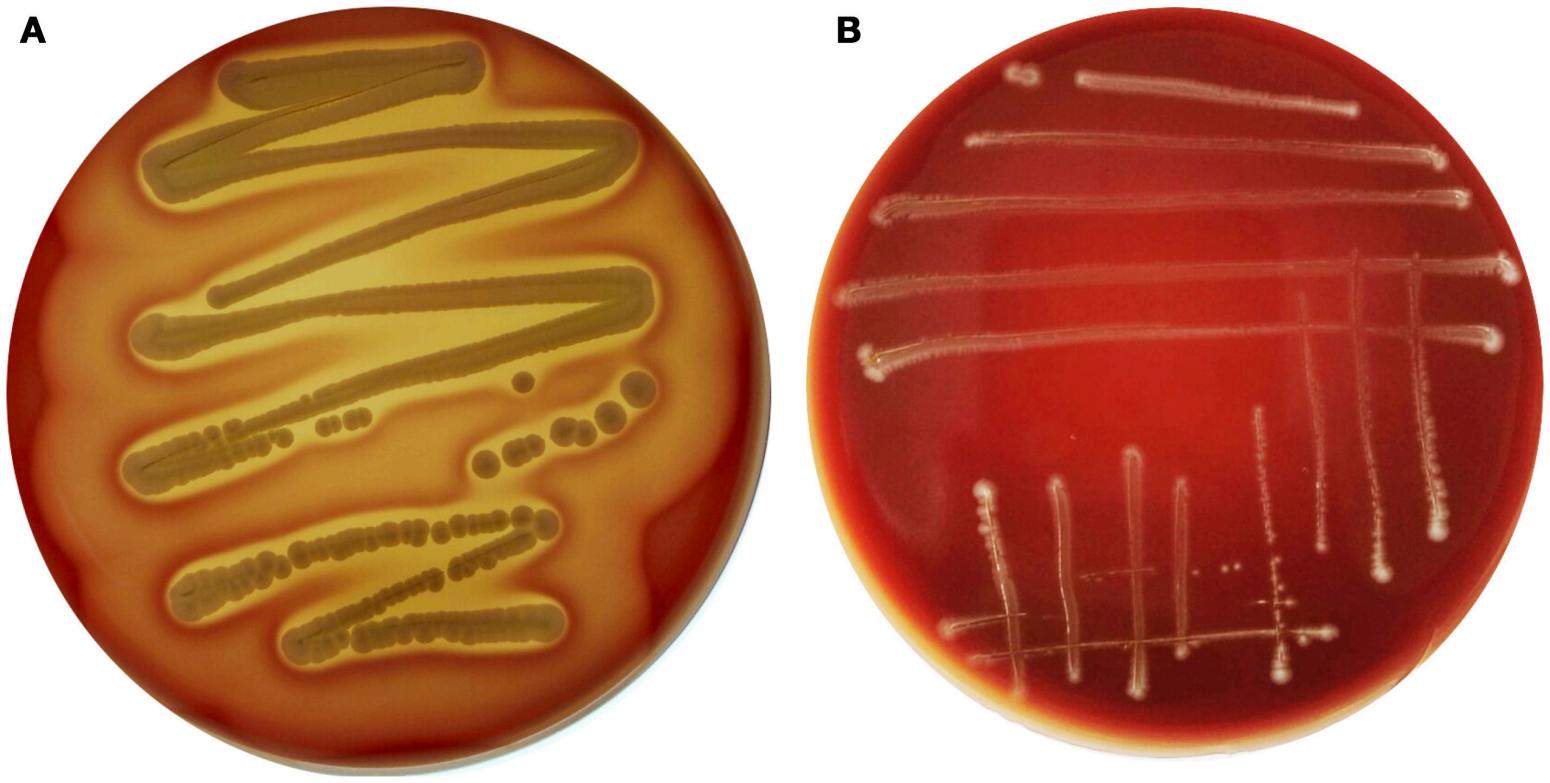
Hemolysin activity of *Enterococcus durans* KLDS6.0930. **(A)** The positive control strain, *Staphylococcus aureus* ATCC 25923, and **(B)** *E. durans* KLDS6.0930.

Interestingly, genes encoding exoenzymes and toxins such as gelatinase (*gelE*), serine protease (*sprE*), hyaluronidase (*hyl*), and cytolysin (*cyl*), which are commonly found in hospital-associated pathogenic strains of this species, were devoid of *E. durans* KLDS6.0930. *In silico* finding for cytolysin was confirmed by a phenotypic assay. After incubation on blood agar plates for 48 h, *E. durans* KLDS6.0930 did not exhibit any hemolytic effects (Figure 3). Notably, *E. durans* KLDS6.0930 also lacks *IS16*, a marker for hospital-associated strains (Werner et al., 2011) and *esp*, a marker of pathogenicity island (van Schaik et al., 2010).

Using Pathogen Finder, a program that performs comparisons between pathogenic and non-pathogenic bacteria using whole genome sequence data, *E. durans* KLDS6.0930 was predicted to be non-pathogen. Combining this result with the virulence factor analysis indicates that *E. durans* KLDS6.0930 is a non-toxigenic strain.

### Putatively harmful metabolites and related determinants

**Figure 4:**
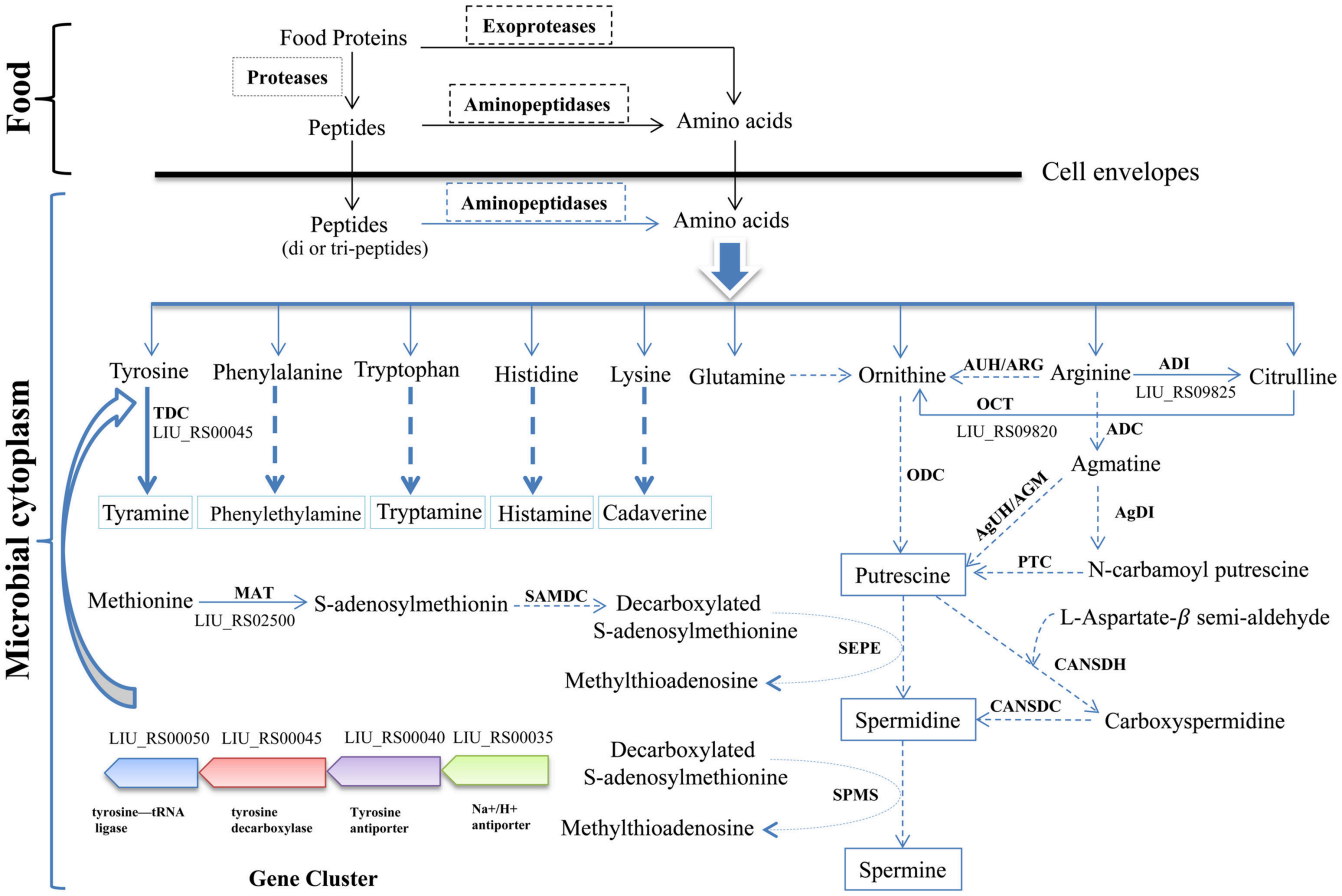
Biosynthesis pathways of different biogenic amines by *Enterococcus durans* KLDS6.0930. Solid arrows represent that the biosynthesis pathways of biogenic amine is effective, correspondingly, the dashed arrows imply invalid; ODC, ornithine decarboxylase; ARG/AUH, arginase/arginine ureohydrolase; ADI, arginine deiminase; ADC, arginine decarboxylase; AgDI, agmatine deiminase; PTC, putrescine transcarbamoylase; AGM/AgUH, agmatinase/agmatine ureohydrolase; OCT, Ornithine carbamoyl transferase; CANSDH, carboxynorspermidine dehydrogenase; CANSDC, carboxynorspermidine decarboxylase; MAT, methionine adenosyl transferase; SAMDC, S-adenosylmethionine decarboxylase; SEPE, spermidine synthase; SPMS, spermine synthase; TDC, tyramine decarboxylase.

The biosynthetic pathway of different biogenic amines is shown in Figure 4. Genes encoding tyramine decarboxylase (TDC) were present in the genome of *E. durans* KLDS6.0930, including a separate TDC gene (LIU_RS02800) and a TDC gene cluster containing a TDC (LIU_RS00045), Na^+^/H^+^ antiporter (LIU_RS00035), tyrosine antiporter (LIU_RS00040), and tyrosine-tRNA ligase (LIU_RS00050).

In addition, polyamines such as putrescine, spermidine, spermine and agmatine are formed via different pathways using ornithine and arginine as the starting substrates (Figure 4). The transformation from arginine to ornithine can occur via two pathways. The first pathway is the direct conversion of ornithine from arginine by arginase/arginine ureohydrolase (ARG/AUH); the responsible gene was not found in the genome of *E. durans* KLDS6.0930. The second pathway is the conversion of arginine into citrulline and followed by catalysis to ornithine via arginine deiminase (ADI) and ornithine carbamoyl transferase (OCT). LIU_RS09825 and LIU_RS09820 encode these two enzymes, respectively, which indirectly catalyze the biosynthesis of ornithine accompanied by the generation of adenosine triphosphate (ATP) (Benkerroum, 2016). However, no genes encoding any of the enzymes for polyamine biosynthesis, except methionine adenosyltransferase (MAT), were found in the genome of *E. durans* KLDS6.0930. Therefore, the *E. durans* KLDS6.0930 genome only possesses the potential for tyramine production. HPLC results further confirmed that tyramine and phenylethylamine were produced by *E. durans* KLDS6.0930, at levels of 51.25 ± 4.38 and 10.48 ± 1.26 mg/L, respectively.

**Figure 5:**
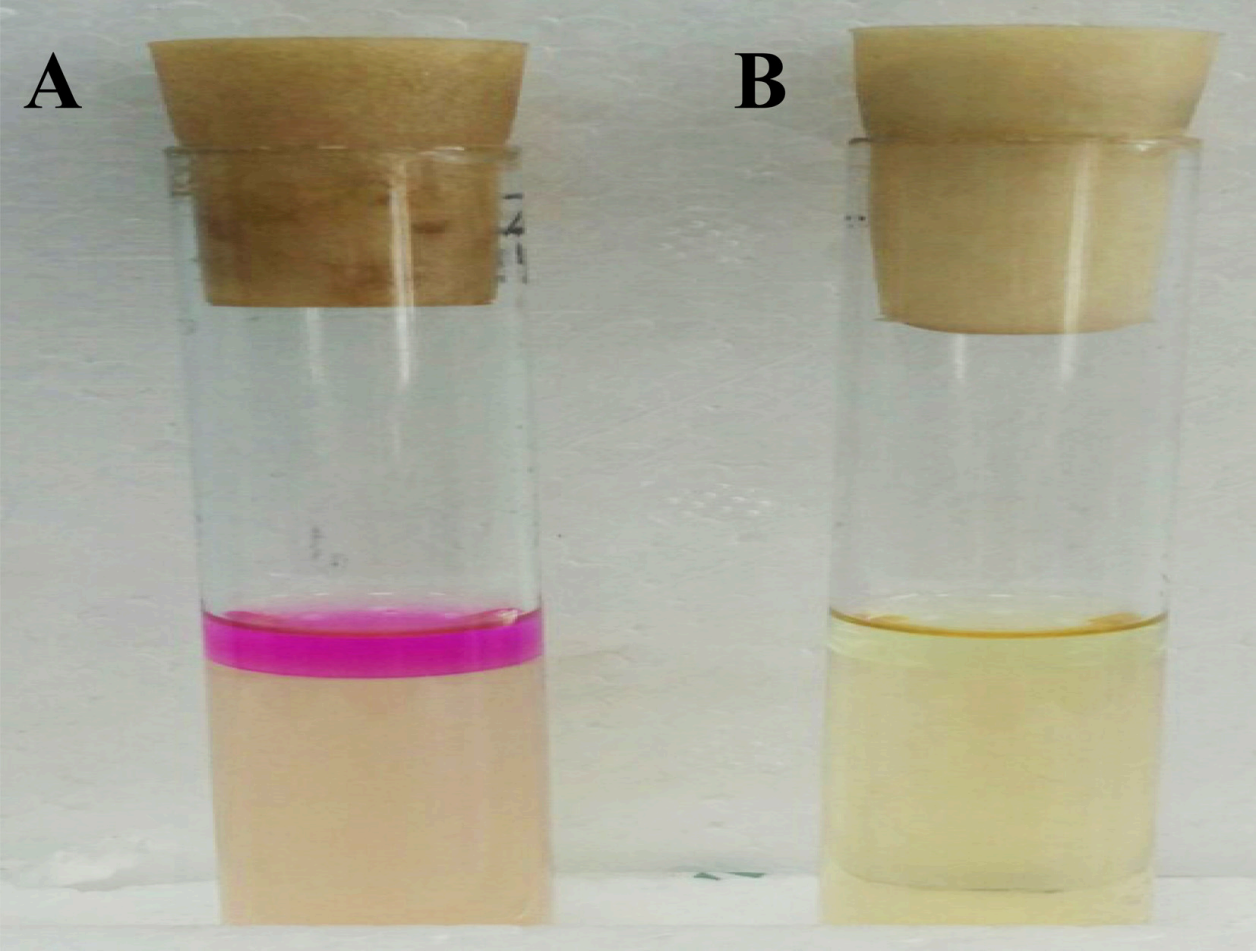
Detection result of the indole produced by *Enterococcus durans* KLDS6.0930. **(A)** The positive control strain, *Escherichia coli ATCC 25922*, and **(B)** *E. durans* KLDS6.0930.

Only two genes (LIU_RS02290 and LIU_RS08060) encoding L-lactate dehydrogenase were identified in the genome of *E. durans* KLDS6.0930; genes encoding D-lactate dehydrogenase were not characterized. The optical purity of the L/D-lactic acid enzymatic assay showed that lactic acid produced by this strain consisted of 100% L-lactic acid. The *E. durans* KLDS6.0930 genome does not harbor any genes related to tryptophan metabolism (Figure S2), which can form indigo. Accordingly, this strain did not generate a red ring in the indigo matrix reaction cultures, compared with the positive control strain *E. coli* ATCC 25922 (Figure 5). LIU_RS10160, LIU_RS07350, and LIU_RS07350, encoding nitroreductase family proteins, were found in the genome of *E. durans* KLDS6.0930, though they were pseudogenes without any function. Correspondingly, no nitroreductase activity was detected in the fermented supernatant.

### Repeated dose 28-day oral toxicity study

#### General health status and body weight changes

General behavior and physical activity of rats were not abnormal for both groups during the 28-days observation period. In terms of weight gain and daily food consumption, there was no statistically significant difference in both genders of the treatment group when compared to the control group (Table S3).

#### Macroscopic examination and histopathology

**Figure 6:**
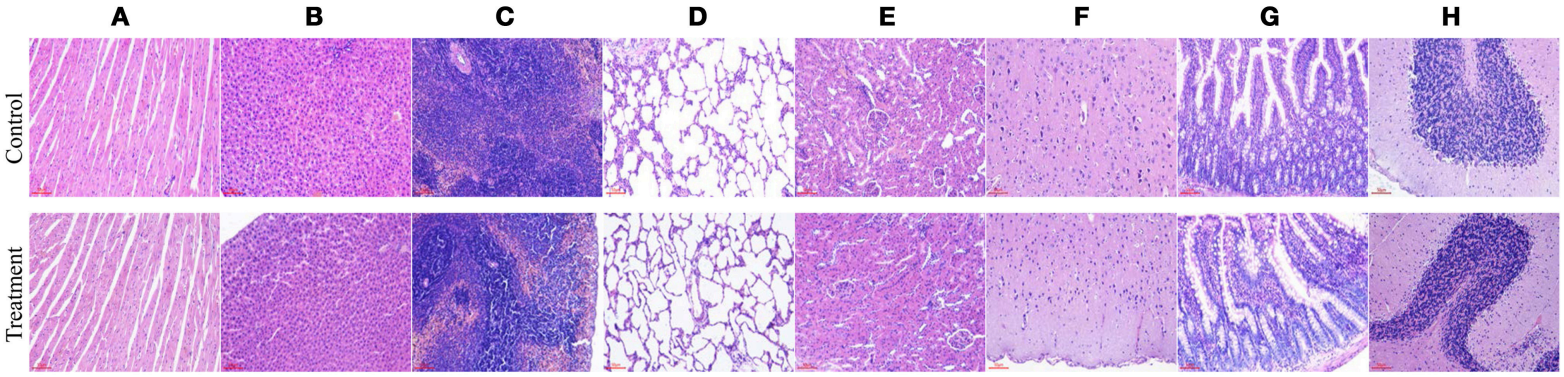
Representative photomicrographs of organs of rats after oral administration of *Enterococcus durans* KLDS6.0930 for 28 days. Control, sterile normal saline; Treatment, 1 × 10^9^ CFU of *E. durans* KLDS6.0930 /kg body weight (BW); **(A)** heart, **(B)** liver, **(C)** spleen, **(D)** lung, **(E)** kidney, **(F)** brain, **(G)** jejunum, **(H)** cerebellum. Bar = 50 μm.

No gross pathological findings were observed in rats of both two groups. Statistical analysis showed that the relative organ weights included heart, liver, spleen, lung, kidney, brain, adrenal, thymus, testis, epididymis, uterus, and ovary were not significantly different between the treatment group and the control group in both sexes (Table 3). Furthermore, light micrograph images of heart, liver, spleen, lung, kidney, brain, and jejunum are presented in Figure 6, there was no obvious histopathological abnormality (inflammation or necrosis) in these examined organs of rats from the treatment group.

**Table 3:** Relative organ weights (%) of male and female rats after oral administration of *Enterococcus durans* KLDS6.0930 for 28 days.

**Group**	**Males**		**Females**	
	**Control**	**Treatment**	**Control**	**Treatment**
Heart	0.335 ± 0.038	0.3401 ± 0.041	0.307 ± 0.033	0.312 ± 0.029
Liver	2.898 ± 0.361	2.912 ± 0.357	3.067 ± 0.277	3.056 ± 0.581
Spleen	0.164 ± 0.047	0.173 ± 0.033	0.204 ± 0.052	0.195 ± 0.043
Lung	0.796 ± 0.143	0.824 ± 0.156	0.636 ± 0.092	0.686 ± 0.175
Kidney	0.368 ± 0.032	0.375 ± 0.036	0.313 ± 0.026	0.309 ± 0.028
Brain	0.562 ± 0.037	0.608 ± 0.049	0.720 ± 0.065	0.737 ± 0.071
Adrenal	0.021 ± 0.004	0.022 ± 0.002	0.028 ± 0.002	0.033 ± 0.005
Thymus	0.027 ± 0.007	0.030 ± 0.009	0.023 ± 0.007	0.026 ± 0.005
Testes	0.476 ± 0.052	0.487 ± 0.046	–	–
Epididymides	0.322 ± 0.017	0.331 ± 0.042	–	–
Uterus	–	–	0.242 ± 0.053	0.249 ± 0.046
Ovaries	–	–	0.062 ± 0.007	0.057 ± 0.005

*Values are presented as mean ± standard deviation (n = 6)*.

*Control, sterile normal saline; Treatment, 1 × 10^9^ CFU of E. durans KLDS6.0930 /kg BW*.

#### Hematology assay and serum biochemistry profile

Hematological and serum biochemistry parameters are reported in Tables 4, 5, respectively. Hematology revealed no significant alterations were found between the treatment and control groups in both sexes. Serum biochemistry parameters showed that administration of *E. durans* KLDS6.0930 also did not result in any treatment-related effects on all rats of the treatment group compared with the control group. In summary, treatment with *E. durans* KLDS6.0930 did not cause any biological deviations outside of the normal ranges.

**Table 4:** Hematological parameters of male and female rats after oral administration of *Enterococcus durans* KLDS6.0930 for 28 days.

**Group**	**Males**		**Females**	
	**Control**	**Treatment**	**Control**	**Treatment**
RBC (× 10^6^/μL)	7.73 ± 0.42	7.83 ± 0.65	7.47 ± 0.45	7.56 ± 0.38
WBC (× 10^3^/μL)	13.37 ± 1.2	13.10 ± 1.72	11.97 ± 1.56	12.47 ± 1.86
HGB (g/L)	148.30 ± 6.05	149.58 ± 6.76	142.57 ± 2.80	142.31 ± 4.45
PLT (× 10^3^/μL)	909.23 ± 101.57	930.80 ± 149.82	*1, 226.67*±213.73	*1, 289.18*±143.72
MCV (fL)	56.03 ± 1.50	56.04 ± 2.21	55.12 ± 1.28	56.39 ± 1.54
MCHC (g/L)	331.43 ± 10.81	332.64 ± 10.88	340.83 ± 5.01	339.23 ± 6.51
Neutrophils (%)	19.47 ± 2.35	21.61 ± 1.76	24.84 ± 4.06	23.82 ± 2.93
Lymphocytes (%)	71.43 ± 5.61	71.79 ± 3.21	73.73 ± 3.12	74.84 ± 4.29
Monocytes (%)	3.83 ± 0.31	3.99 ± 0.63	5.17 ± 0.45	5.02 ± 0.85
Eosinophils (%)	1.40 ± 0.44	1.26 ± 0.29	1.30 ± 0.61	1.17 ± 0.37

*Values are presented as mean ± standard deviation (n = 6)*.

*Control, sterile normal saline; Treatment, 1 × 10^9^ CFU of E. durans KLDS6.0930 /kg BW; RBC, red blood cell count; WBC, white blood cell count; HGB, hemoglobin; PLT, platelet count; MCV, mean corpuscular volume; MCHC, mean corpuscular hemoglobin concentration*.

**Table 5:** Serum biochemical parameters of male and female rats after oral administration of *Enterococcus durans* KLDS6.0930 for 28 days.

**Group**	**Males**		**Females**	
	**Control**	**Treatment**	**Control**	**Treatment**
AST (U/L)	148.80 ± 5.99	149.56 ± 16.03	91.27 ± 8.75	93.25 ± 10.67
ALT (U/L)	72.77 ± 8.88	72.59 ± 4.24	52.53 ± 7.36	54.41 ± 6.52
ALP (U/L)	159.33 ± 9.29	163.24 ± 10.81	88.33 ± 8.50	89.72 ± 9.73
TBIL (μmol/L)	1.00 ± 0.36	0.96 ± 0.21	1.03 ± 0.21	1.00 ± 0.25
TP (g/L)	53.33 ± 3.68	53.64 ± 5.16	67.77 ± 4.50	68.47 ± 5.16
ALB (g/L)	42.13 ± 3.44	39.55 ± 3.79	42.93 ± 2.60	44.61 ± 4.27
GLU (mmol/L)	5.86 ± 1.60	5.97 ± 0.91	8.97 ± 0.87	8.62 ± 0.89
TG (mmol/L)	0.48 ± 0.05	0.49 ± 0.10	0.51 ± 0.02	0.53 ± 0.05
TC (mmol/L)	1.42 ± 0.11	1.45 ± 0.22	2.23 ± 0.25	2.19 ± 0.36
HDL (mmol/L)	1.68 ± 0.25	1.65 ± 0.14	1.77 ± 0.31	1.83 ± 0.26
LDL (mmol/L)	0.21 ± 0.09	0.20 ± 0.08	0.33 ± 0.08	0.32 ± 0.07
UREA (mmol/L)	6.47 ± 1.32	6.81 ± 0.72	6.63 ± 0.38	6.54 ± 0.32
CRE (μmol/L)	52.70 ± 4.93	54.62 ± 5.47	73.17 ± 2.35	71.28 ± 2.84
Na (mmol/L)	140.93 ± 2.35	143.06 ± 2.61	142.17 ± 3.25	143.36 ± 4.29
Cl (mmol/L)	105.40 ± 2.33	102.35 ± 2.68	100.67 ± 2.37	99.21 ± 3.11
Ca (mmol/L)	2.69 ± 0.13	2.45 ± 0.35	2.56 ± 0.07	2.51 ± 0.08
P (mmol/L)	3.12 ± 0.30	2.96 ± 0.27	2.48 ± 0.07	2.62 ± 0.05

*Values are presented as mean ± standard deviation (n = 6)*.

*Control, sterile normal saline; Treatment, 1 × 10^9^ CFU of E. durans KLDS6.0930 /kg BW; AST, aspartate aminotransferase; ALT, alanine aminotransferase; ALP, alkaline phosphatase; TBIL, total bilirubin, TP, total protein; ALB, albumin; GLU, glucose; TG, triglycerides; TC, total cholesterol; HDL, high density lipoprotein cholesterol; LDL, low density lipoprotein cholesterol; UREA, urea; CRE, creatinine; Na, sodium; Cl, chloride; Ca, calcium; P, inorganic phosphorus*.

#### Cecal microbiota

**Figure 7:**
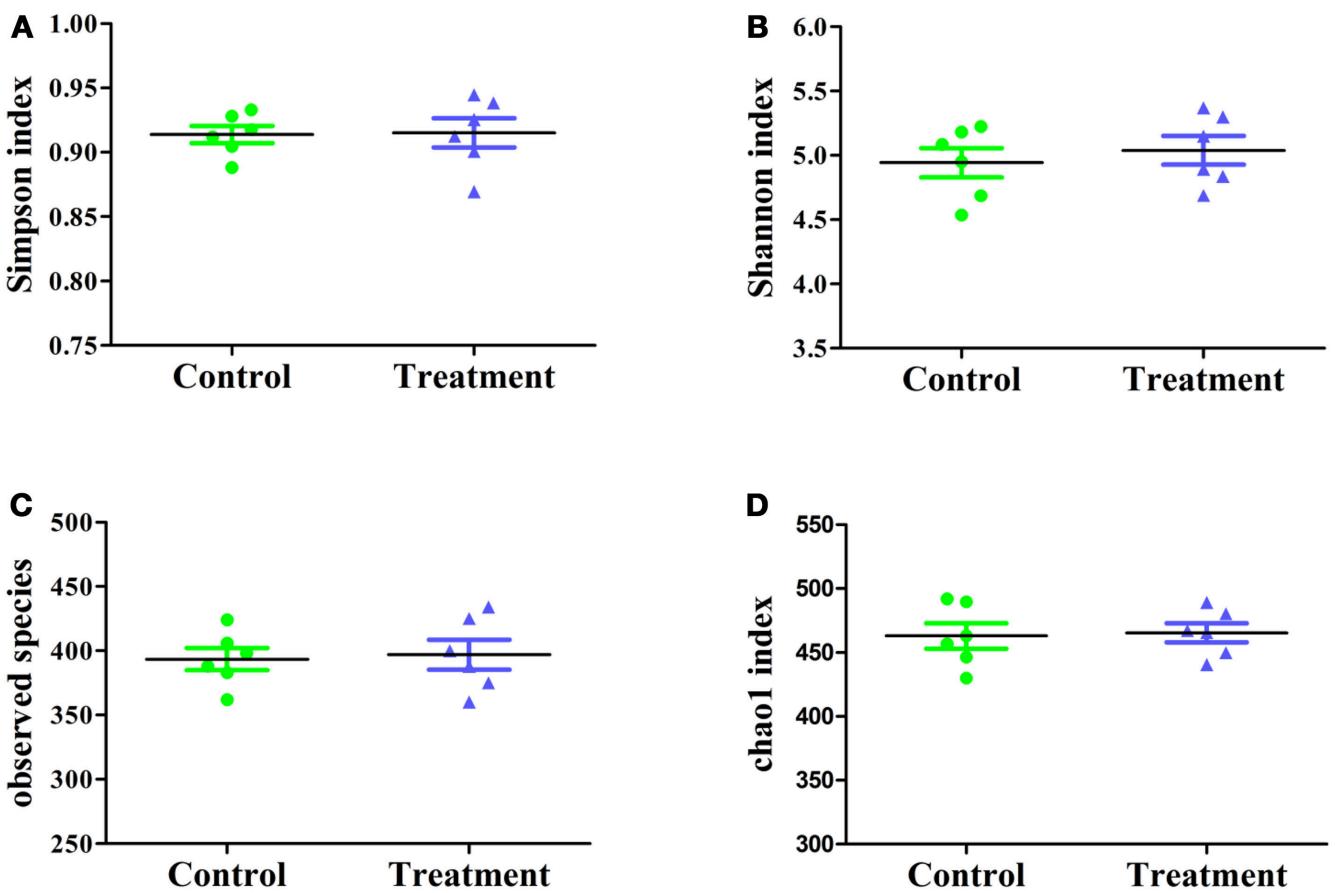
Cecal microbiota α-diversity in male rats after oral administration of *Enterococcus durans* KLDS6.0930 for 28 days. Control, sterile normal saline; Treatment, 1 × 10^9^ CFU of *E. durans* KLDS6.0930 /kg BW. **(A)** Simpson index, **(B)** Shannon index, **(C)** observed species, **(D)** Chao1 index.

The effect of *E. durans* KLDS6.0930 on cecal microbial diversity was determined by amplicon sequencing and analyzed by α-diversity and β-diversity. There were no significant differences in α-diversity, which include Simpson index, Shannon index, observed species and Chao1 index (Figure 7) between the treatment and control groups in male rats. This implied that *E. durans* KLDS6.0930 treatment did not change the diversity and abundance of cecal microbiota of male rats.

**Figure 8:**
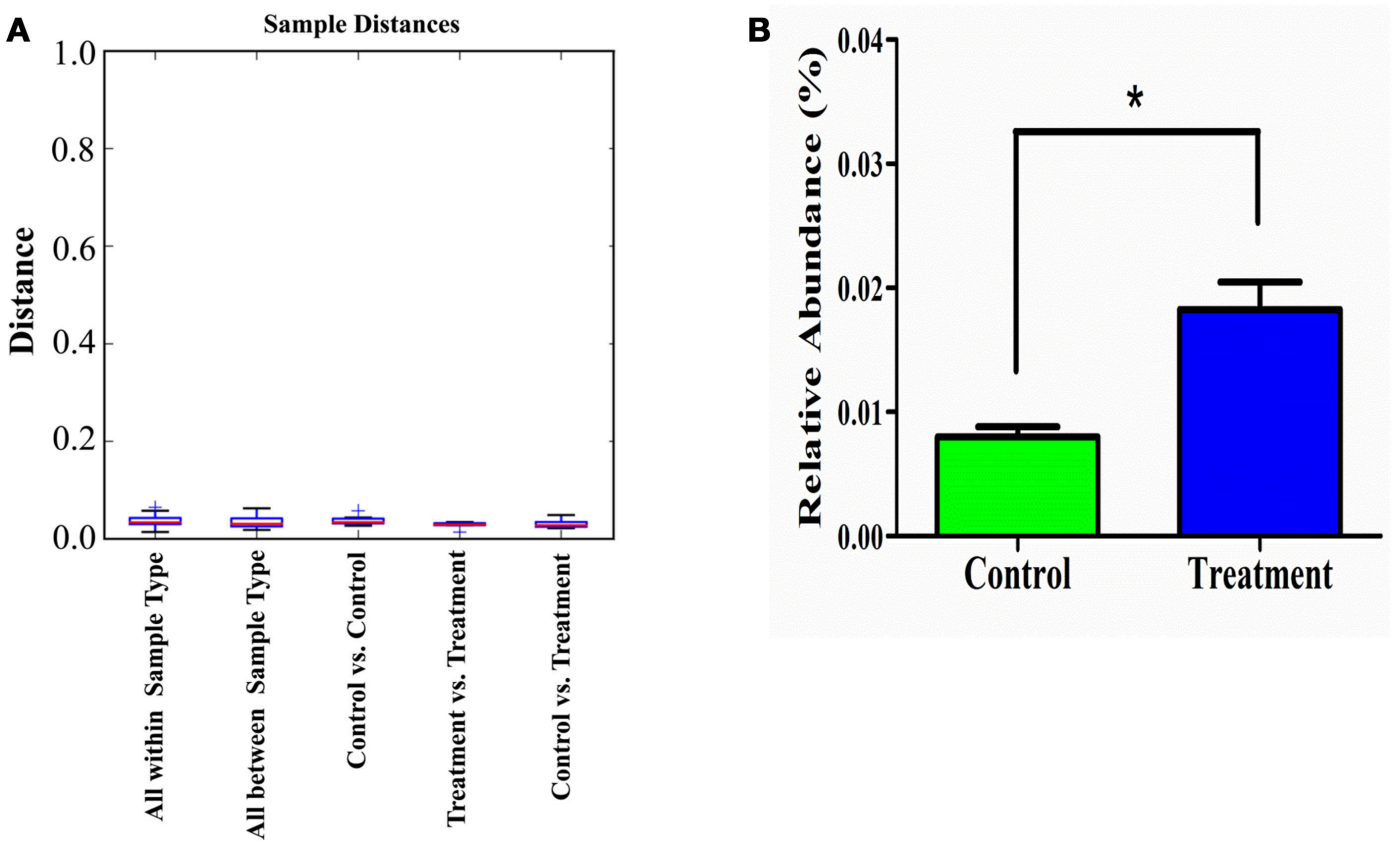
Weighted unifrac distance (β-diversity) of the cecal microbiota **(A)** and relative abundance of *Enterococcus* **(B)** in male rats after oral administration of *Enterococcus durans* KLDS6.0930 for 28 days. Control, sterile normal saline; Treatment, 1 × 10^9^ CFU of *E. durans* KLDS6.0930 /kg BW. Statistical analysis was conducted by unpaired two-tailed student's t-test. *P < 0.05.

Additionally, the sample distance based on weighted UniFrac analysis is shown in Figure 8. The differences in the inter-class distances of the treatment and control groups were not significant. Moreover, the distance between all samples was close, indicating that the differences in cecal microbial β-diversity of the treatment and control groups of male rats was very small. This agrees with the results for α-diversity, suggesting the *E. durans* KLDS6.0930 treatment did not disrupt the normal cecal microbiota. Similar results from both groups in female rats were obtained as well (data not shown). Therefore, changes in cecal microbiota due to *E. durans* KLDS6.0930 treatment are not harmful.

As shown in Figure 8, *E. durans* KLDS6.0930 administration specifically enriched the abundance of enterococci in treated rats, indirectly indicating *E. durans* KLDS6.0930 was present in the cecal content. Furthermore, we used cFDA-SE to trace *E. durans* KLDS6.0930 in the cecum (Figure S3). One day after gavage, the results from flow cytometry confirmed that *E. durans* KLDS6.0930 labeled with cFDA-SE was detectable in the cecum of rats, collating our previous results that *E. durans* KLDS6.0930 is highly tolerant to acid and bile and adherent to Caco-2 cells *in vitro* via the necessary genetic elements (Guo et al., 2016; Liu et al., 2016). These findings indicate that *E. durans* KLDS6.0930 can survive and colonize the cecum.

## Discussion

Enterococci have gained notoriety over the past few decades as leading nosocomial pathogens, but have recently been investigated as potential probiotic agents (Ogier and Serror, 2008). Concerns about the use of enterococci as a double-edged sword have led to an increasing number of studies intended to evaluate the safety of certain enterococci strains (Kopit et al., 2014; Zhang et al., 2016; Peng et al., 2017).

Our previous studies suggest that *E. durans* KLDS6.0930 may be a potential probiotic strain. According to the FAO/WHO, probiotic microorganisms must be non-pathogenic and non-toxic (FAO/WHO, 2012). In this study, *E. durans* KLDS6.0930 was exhaustively analyzed for safety using multiple methods.

*E. durans* KLDS6.0930 carries potential CRISPR arrays and Type II-A CRISPR associated enzymes. A previous study showed that CRISPR serves as a formidable defense against phage invasion as well as restricting the dissemination of antibiotic resistance genes (Marraffini and Sontheimer, 2008). In addition, *E. durans* KLDS6.0930 also contains R-M systems, providing another form of genome defense through self-recognition vs. non-self-recognition of methylation signatures (Price et al., 2016). Supporting these, only one prophage was found in the genome of *E. durans* KLDS6.0930. In addition, no functional genes for the portal protein, one of the essential phage proteins, were found, suggesting that the prophage was defective (Isidro et al., 2004). These defense systems may play key roles in the stability of the *E. durans* KLDS6.0930 genome.

*E. durans* KLDS6.0930 lacks the established markers associated with the clinical strains, including *esp, hyl*, and *IS16*, which were specified by the EFSA as targets for the safety evaluation of *E. faecium* strains intended as additives for animal feed (EFSA, 2012b). On the other hand, enterococci that contain a type II-A CRISPR system are reported to be non-pathogenic (Palmer and Gilmore, 2010). As noted above, *E. durans* KLDS6.0930 carries a type II-A CRISPR, further supporting its non-pathogenicity.

Emerging evidence indicates that human intestinal bacteria can be a reservoir for antibiotic resistance genes that can be transferred to human pathogens. For a bacterial strain to qualify as a probiotic candidate, its susceptibility or resistance to a range of antibiotics must be assessed (EFSA, 2012a). Antibiotic resistance assays showed that *E. durans* KLDS6.0930 was resistant to aminoglycosides, clindamycin, and rifampicin, and genes associated with resistance to aminoglycosides, clindamycin, and rifampin resistance were detected. The resistance profile of *E. durans* KLDS6.0930 requires further investigation to determine the nature of the antibiotic resistance (intrinsic resistance or acquired resistance). According to the present EFSA guidelines on the characterization of microbiological feed additives (EFSA, 2018), a non-intrinsic resistance combined with the presence of a resistance-encoding gene disqualifies the strain as a feed additive in the European Union (EU). Initially, it may be necessary to distinguish between acquired and intrinsic resistances when there is limited information on MICs within the relevant taxonomical unit (EFSA, 2012a). In this case, the structural nature and genetic basis of the antibiotic resistance must be demonstrated by analyzing a representative selection of strains belonging to that taxonomical unit. As there are limited data on antibiotic resistances of *E. durans*, we analyzed the genetic basis of antibiotic resistances in *E. durans* KLDS6.0930 genome by comparing it with eight other sequenced *E. durans* genomes. Table S4 shows that genes associated with resistance to aminoglycosides, clindamycin and rifampin were identified in all analyzed *E. durans* genomes. Furthermore, no mobile elements, such as transposases or insertion sequences, were found in the flanking regions of these antibiotic resistance genes. This implied that these antibiotic resistance genes are ubiquitous in the genome of *E. durans*, meaning that resistances to aminoglycosides, clindamycin, and rifampin are intrinsic resistances. However, these antibiotic resistances of all other *E. durans* strains should be further investigated.

Generally, the phenotype does not completely reflect the genotype. Enterococcal isolates are often resistant to β-lactam antibiotics, likely due to the expression of low-affinity penicillin-binding protein 5 (PBP5) (Fontana et al., 1996). Four genes encoding PBPs (LIU_RS05885, LIU_RS07610, LIU_RS08470, LIU_RS10805) were found in *E. durans* KLDS6.0930 genome. The genes are annotated as PBP2b, PBP1b, PBP1a, and PBP2x, and have a low identity with PBP5 according to BLASTP. Despite the presence of these PBPs, however, *E. durans* KLDS6.0930 is sensitive to penicillin and ampicillin, a phenomenon also observed in *E. hirae* R17 (Peng et al., 2017).

*E. durans* KLDS6.0930 was sensitive to trimethoprim but contained the trimethoprim resistance gene (LIU_RS06485). Interestingly, the MIC value of tetracycline for *E. durans* KLDS6.0930 was equal to the cut-off value for *Enterococcus* species. Based on EFSA guidelines (EFSA, 2012a), strains are considered susceptible when the MIC value is equal to or lower than the reference cut-off value. Therefore, *E. durans* KLDS6.0930 is susceptible to tetracycline, nevertheless, two tetracycline resistance genes were found in the genome. Expression of these genes may have been sufficiently low or induced only under specific conditions, such as environmental stimuli or signals. Additional studies are needed to resolve this discrepancy, as differences between the antibiotic resistance phenotype and genotype may result from modification of genes identified *in silico* after transcription, pseudogenes, or misidentification due to only partial similarities to known resistance genes. The presence of these resistance genes, therefore, does not represent a dangerous characteristic of the bacterium.

Biogenic amines have a strong impact on maintenance of normal physiological function of the human body. Nonetheless, they may be hazardous to human health if their levels in foods or beverages reach a critical threshold (EFSA, 2011). Therefore, it is essential to evaluate enterococcal strains for their ability to produce biogenic amines. Genes for biogenic amine production were generally absent based on the genomic data of *E. durans* KLDS6.0930, except those encoding tyramine. Tyramine, phenylethylamine, histamine, tryptamine, and cadaverine are directly catalyzed by amino acid decarboxylases from their respective precursor amino acids (Benkerroum, 2016). Two non-homologous TDC-coding genes were found in the *E. durans* KLDS6.0930 genome. It is likely that these genes confer the potential of tyramine biosynthesis to the strain. Tyramine, together with tryptamine and phenylethylamine, are classified as vasoactive amines, as high levels can provoke dangerous hypertension (Mohedano et al., 2014). Fortunately, HPLC showed that *E. durans* KLDS6.0930 produced 51.25 ± 4.38 mg/L tyramine, lower than the toxic level of tyramine (100–800 mg/kg) in foods (ten Brink et al., 1990).

Usually, phenylethylamine production is associated with tyramine production, as demonstrated for some Enterococci (Bover-Cid et al., 2001). *E. durans* KLDS6.0930 also produced 10.48 ± 1.26 mg/L phenethylamine, under the threshold value of 30 mg/kg (ten Brink et al., 1990). However, *E. durans* KLDS6.0930 was not found to encode any of the genes related to phenethylamine. A previous study showed that a decarboxylase can decarboxylate different structural homologs (Benkerroum, 2016). Here, the TDC of *Enterococcus* can accept tyrosine and phenylalanine as substrates (Marcobal et al., 2006, 2012; EFSA, 2011), as they are aromatic amino acids with similar structures.

As a part of extensive safety assessments, *in vivo* studies can provide valuable information. The physiology of rats behaves similarly in some situations to that of humans, making rats a suitable model for toxicity studies. A 28-day repeated oral dose toxicity study was performed to assess the safety of *E. durans* KLDS6.0930 in a rat model. No significant strain-related toxigenic symptoms in the physical appearance of male or female rats were observed in any of the groups. Changes in food consumption, BW and organ weight are considered indicators of toxic effects of a test material. Hematological parameters are commonly used to detect inflammation and infections. Serum biochemical parameters are usually used to determine of organ-related problems. There were no significant differences in food consumption, BW, organ weight, serum biochemical parameters, or hematological parameters between the control and treatment groups, implying that *E. durans* KLDS6.0930 administration for 28 days did not cause any adverse effects. Histopathology showed that there were no anomalies or changes related to repeated oral application of *E. durans* KLDS6.0930 for 28 days. Notably, our results suggested that a daily dose of 1 × 10^9^ CFU of *E. durans* KLDS6.0930 /kg BW daily intake was safe and did not give rise to any treatment-related toxicity effects.

To support host health, cecal bacterial populations should be analyzed when evaluating the safety of probiotics. Parisa et al. (2016) showed that *Lactobacillus* strains decreased pathogenic bacterial populations such as *Salmonella* and *E. coli* while increasing beneficial bacterial populations such as lactobacilli and bifidobacteria in the cecal contents of rats through the colony counting method (Parisa et al., 2016). Mukerji et al. (2016) revealed that AB-LIFE® increased *L. plantarum* counts in a dose-dependent manner by qPCR method. However, these methods focus on individual gut microbes and are not nearly as extensive and comprehensive enough when compared to next-generation sequencing. The effect of *E. durans* KLDS6.0930 on cecal microbiota diversity was evaluated by sequencing the 16S rRNA V4 region. No significant differences in α-diversity and β-diversity were detected between the control and treatment groups, indicating that *E. durans* KLDS6.0930 did not impact cecal microbiota diversity and abundance in healthy male and female rats. This result is consistent with our previous study (Li B. et al., 2017), indicating that *E. durans* KLDS6.0930 administration is safely modulates gut microbiota without causing detrimental effects on diversity.

In summary, we present a safety assessment of *E. durans* KLDS6.0930 using multiple methods. The results *in vitro* demonstrated that *E. durans* KLDS6.0930 does not raise safety concerns regarding acquired antibiotic resistance genes, virulence factors, D-lactic acid, indole production, or nitroreductase activity, as supported by phenotypic and genomic data. The production levels of tyramine and phenethylamine were found to be well below established limits to cause harm to consumers. Furthermore, the *in vivo* studies showed that *E. durans* KLDS6.0930 is not toxic to male or female rats. These findings confirm that *E. durans* KLDS6.0930 is a non-pathogenic strain and can be used as a potential probiotic for human consumption and animal feed.

## Author contributions

GH and BL designed the study. BL, MZ, DJ, SC, and SS performed experiments. BL, FL, MZ, LZ, and SE analyzed data. SE, BL, SC, and SS wrote manuscript.

### Conflict of interest statement

The authors declare that the research was conducted in the absence of any commercial or financial relationships that could be construed as a potential conflict of interest.
