# Supplementary material for: Evaluating the Safety of Potential Probiotic Enterococcus durans KLDS6.0930 Using Whole Genome Sequencing and Oral Toxicity Study
Source: Front Microbiol. 2018 Aug 21;9:1943. doi: 10.3389/fmicb.2018.01943 (PMC6110905; doi:10.3389/fmicb.2018.01943)
Supplement: Supplementary file 1 [file Table_1.DOCX]

**Table S1.** Putative virulence factors in *Enterococcus durans* KLDS6.0930 genome by searching with the virulence factor database (VFDB).

| Gene | Gene ID | Function | Identity (%) | E-value | Match (%) |
| --- | --- | --- | --- | --- | --- |
| *efaA* | LIU_RS03220 | endocarditis specific antigen (VF0354) | 62.68 | 2.00E-126 | 88.745 |
| *efaA* | LIU_RS03820 | periplasmic solute binding protein (CVF610) | 75.51 | 1.00E-167 | 94.838 |
| *CDS1* | LIU_RS09775 | phosphatidate cytidylyltransferase [Capsule (CVF618)] | 85.34 | 4.00E-154 | 100 |
| *uppS* | LIU_RS09780 | undecaprenyl diphosphate synthase [Capsule (CVF618)] | 89.63 | 0 | 100 |
| *galE* | LIU_RS09890 | UDP-glucose 4-epimerase [Capsule (CVF567)] | 62.5 | 3.00E-157 | 99.696 |
| *galU* | LIU_RS12185 | glucose-1-phosphate uridylyltransferase [Capsule (CVF186)] | 72.26 | 6.00E-154 | 100 |
| *rfbA-1* | LIU_RS05410 | glucose-1-phosphate thymidylyltransferase [Capsule (CVF186)] | 80.56 | 2.00E-177 | 100 |
| *rmlB* | LIU_RS05420 | putative dTDP-glucose-4,6-dehydratase [Capsule (CVF186)] | 83.38 | 0 | 95.029 |
| *strL* | LIU_RS05425 | dTDP-4-dehydrorhamnose reductase [Capsule (CVF186)] | 63.9 | 1.00E-125 | 98.226 |
| *hasC* | LIU_RS10850 | UDP-glucose pyrophosphorylase [Capsule (CVF186)] | 75.77 | 3.00E-164 | 93.312 |
| *srtC* | LIU_RS08105 | putative pilus-dedicated sortase [PilB-type pili (PGS3) ] | 73.45 | 3.00E-120 | 59.946 |
| *ebpC* | LIU_RS08110 | PilB-type pili sununit | 73.05 | 0 | 99.837 |
| *ebpB* | LIU_RS08115 | PilB-type pili subunit | 51% | 5.00E-173 | 100% |
| *ebpA* | LIU_RS08120 | PilB-type pili subunit | 69% | 0 | 57% |
| *ebpR* | LIU_RS08125^ψ^ | Regulatory gene for enterococcus biofilm and pilus (ebp) |  |  |  |
| *ebp* | LIU_RS15130 | Endocarditis and Biofilm-Associated Pilus subunitA | 66.88 | 0 | 94.065 |
| *pilA* | LIU_RS13970^P^ | major pilin subunit (PGS1, pilin gene clusters 1) (AI132)] | 98.94 | 0 | 100 |
| *pilE* | LIU_RS13960^P^ | [PilA-type pili (PGS1, pilin gene clusters 1)] | 99.6 | 3.00E-144 | 100 |
| *pilF* | LIU_RS13945^P^ | [PilA-type pili (PGS1, pilin gene clusters 1) (AI132)] | 99.43 | 0 | 100 |
| *srtC* | LIU_RS13975^P^ | [PilA-type pili (PGS1, pilin gene clusters 1)] | 99.1 | 2.00E-165 | 100 |
| *srtC* | LIU_RS13965^P^ | [PilA-type pili (PGS1, pilin gene clusters 1)] | 99.15 | 2.00E-156 | 93.6 |
| *srtC* | LIU_RS13955^P^ | [PilA-type pili (PGS1, pilin gene clusters 1)] | 98.67 | 2.00E-48 | 87.209 |
| *srt2* | LIU_RS13845^P^ | biofilm enhancer in enterococci (AI134) | 97.22 | 0 | 67.56 |
| *srt1* | LIU_RS13850^P^ | biofilm enhancer in enterococci (AI134) | 98.49 | 0 | 100 |
| *bee1* | LIU_RS13865^P^ | biofilm enhancer in enterococci (AI134) | 97.69 | 0 | 100 |
| *bee3* | LIU_RS13855^P^ | biofilm enhancer in enterococci (AI134) | 97.37 | 0 | 100 |
| *bee2* | LIU_RS13860^P^ | biofilm enhancer in enterococci (AI134) | 99.59 | 2.00E-150 | 100 |
| *bopD* | LIU_RS00030 | LacI family sugar-binding transcriptional regulator (CVF615) | 89.64 | 0 | 99.705 |
| *lisR* | LIU_RS05970 | two-component response regulator (CVF253) | 78.95 | 4.00E-116 | 100 |
| *tig* | LIU_RS09445 | trigger factor (CVF149) | 66.67 | 0 | 99.532 |
| *sigA* | LIU_RS06305 | RNA polymerase sigma factor SigA (CVF325) | 62.71 | 6.00E-115 | 82.113 |
| *tuf* | LIU_RS06260 | elongation factor Tu (CVF587) | 72.34 | 0 | 99.746 |
| *tuf* | LIU_RS01060 | translation elongation factor Tu (CVF587) | 75.63 | 0 | 99.746 |
| *dnaK* | LIU_RS09560 | molecular chaperone DnaK (AI392) | 64.81 | 0 | 88.524 |
| *groEL* | LIU_RS12590 | chaperonin GroEL (CVF403) | 71.9 | 0 | 95.378 |
| *clpC* | LIU_RS01460 | endopeptidase Clp ATP-binding chain C (VF0072) | 60.52 | 0 | 97.82 |
| *clpE* | LIU_RS05695 | ATP-dependent protease (VF0073) | 60.85 | 0 | 98.787 |
| *clpP* | LIU_RS11815 | ATP-dependent Clp protease proteolytic subunit (VF0074) | 80.61 | 5.00E-119 | 99.492 |
| *eno* | LIU_RS11130 | phosphopyruvate hydratase (CVF153) | 83.14 | 0 | 100 |
| *bsh* | LIU_RS08420 | Bile-salt hydrolase (CVF250) | 71.3 | 0 | 100 |
| *lap* | LIU_RS00250 | aldehyde-alcohol dehydrogenase protein (CVF228) | 69.52 | 0 | 99.768 |
| *sugC* | LIU_RS12265 | Trehalose-recycling ABC transporter (CVF651) | 61.15 | 3.00E-121 | 80.216 |
| *slrA* | LIU_RS07470 | lipoprotein rotamase A (CVF129) | 62.09 | 2.00E-66 | 72.8 |
| *gapA* | LIU_RS11145 | glyceraldehyde-3-phosphate dehydrogenase | 79.88 | 0 | 100 |
| *msrA* | LIU_RS00600 | peptide methionine sulfoxide reductase msrA/msrB | 63.28 | 1.00E-54 | 87.671 |
| *lplA1* | LIU_RS05030 | putative lipoate protein ligase A (CVF238) | 62.39 | 1.00E-152 | 100 |
| *sprE* | ND | serine protease |  |  |  |
| *fsr* | ND | Quorum sensing system |  |  |  |
| *cyl* | ND | Cytolysin |  |  |  |
| *esp* | ND | Enterococcal surface protein |  |  |  |
| *gelE* | ND | Gelatinase |  |  |  |
| *hyl* | ND | Hyaluronidase |  |  |  |
| *IS16* | ND | Mobile insertion sequence |  |  |  |

^ψ^, pseudogene; ^P^,plasmid; and ND, not detected.

**Table S2.** Biofilm formation ability of *Enterococcus durans* KLDS6.0930.

| Strain | OD_595_ | ODc | Criteria | Biofilm formation |
| --- | --- | --- | --- | --- |
| *E. durans* KLDS6.0930 | 0.19 ± 0.02 | 0.14 | 2ODc > OD_595_ > ODc | + |
| S. aureus ATCC 25923 | 0.64 ± 0.03 | 0.14 | OD_595_ > 4ODc | +++ |

ODc, cut-off OD; +++, strong biofilm producer; ++, moderate biofilm producer; +, weak biofilm producer.

**Table S3.** Body weight and food data of male and female rats after oral administration of *Enterococcus durans* KLDS6.0930 for 28 days.

| Group | Males | | Females | |
| --- | --- | --- | --- | --- |
|  | Control | Treatment | Control | Treatment |
| Body weight gain (g) | 69.9±6.7 | 71.2±6.3 | 32.0±2.7 | 33.8±3.0 |
| Daily Food consumption (g) | 24.1±0.9 | 25.1±1.2 | 16.9±1.2 | 17.4±1.1 |

Values are presented as mean ± standard deviation (n=6).

Control, sterile normal saline; Treatment, 1 × 10^9^ CFU of *E. durans* KLDS6.0930 /kg BW.

**Table S4.** The distribution of genes associated with aminoglycosides, clindamycin and rifampicin resistance in the genomes of all sequenced *Enterococcus durans* strains.

| Strain | | Identity of homolog (%) | | | |
| --- | --- | --- | --- | --- | --- |
| KLDS6.0930 | LIU_RS02535 | | LIU_RS02530 | LIU_RS01575 | LIU_RS12375 |
| KLDS6.0933 | 100 | | 100 | 100 | 100 |
| BDGP3 | 99 | | 100 | 99 | 100 |
| ATCC 6056 | 99 | | 99 | 100 | 99 |
| IPLA 655 | 100 | | 100 | 100 | 100 |
| F0321E 104 | 99 | | 100 | 99 | 100 |
| FDAARGOS_396 | 99 | | 99 | 99 | 99 |
| CVM N59549F | 99 | | 99 | 99 | 98 |
