# Supplementary material for: Evaluating the Safety of Potential Probiotic Enterococcus durans KLDS6.0930 Using Whole Genome Sequencing and Oral Toxicity Study
Source: Front Microbiol. 2018 Aug 21;9:1943. doi: 10.3389/fmicb.2018.01943 (PMC6110905; doi:10.3389/fmicb.2018.01943)
Supplement: Supplementary file 2 [file Image_1.pdf]

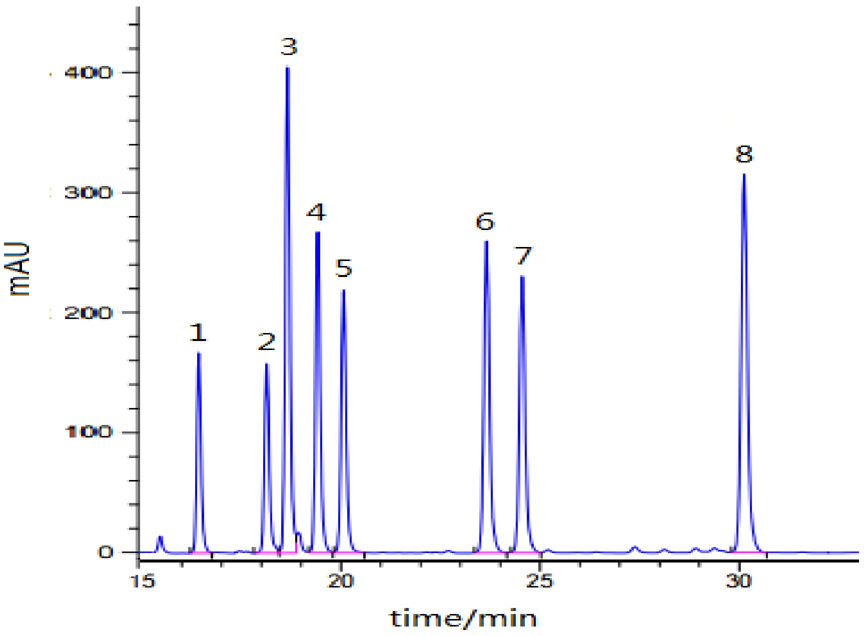

Figure S1. Typical HPLC chromatograms of biogenic amines in standard solution

1, tryptamine; 2, phenylethylamine; 3, putrescine; 4, cadaverine; 5, histamine; 6, tyramine; 7, spermidine; 8,spermine.

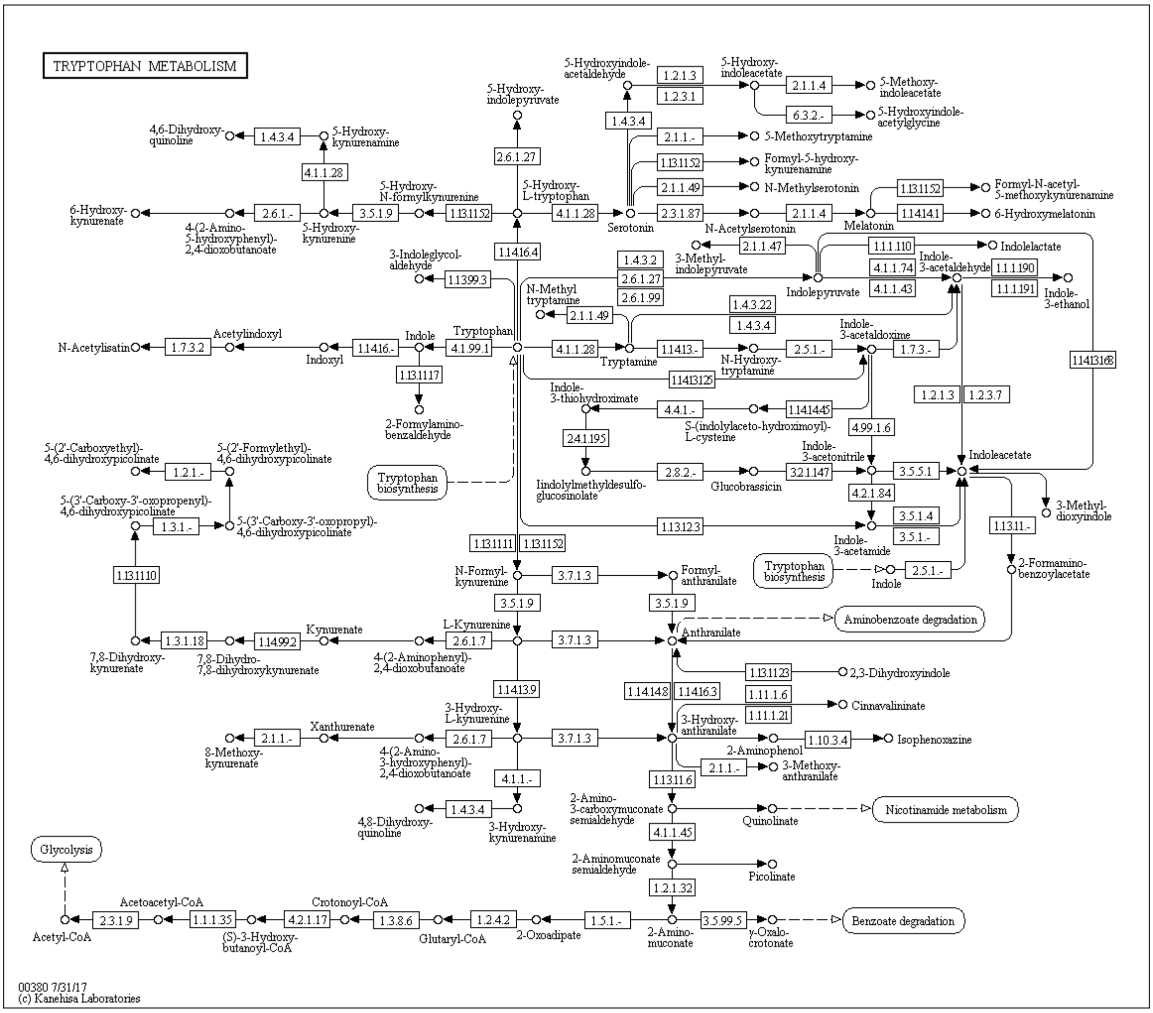

Figure S2. The pathway of tryptophan metabolism and indole biosynthesis by *Enterococcus durans* KLDS6.0930

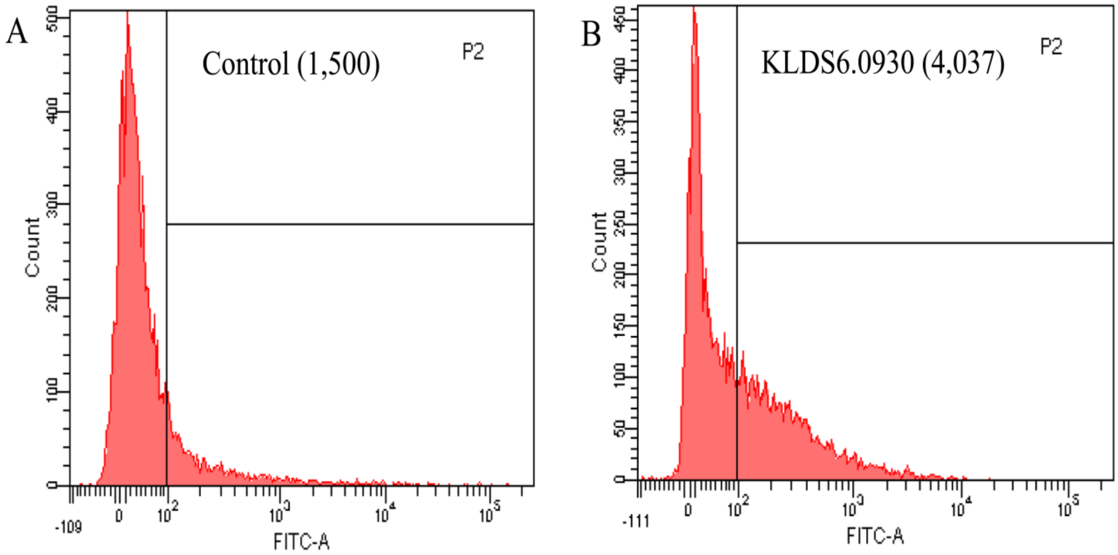

Figure S3. Representative flow cytometry images of *Enterococcus durans* KLDS6.0930 labeled with carboxyfluorescein diacetate succinimidyl ester (cFDA-SE) in the cecum of rats after administration for 1 day. Control, 1 mL of sterile normal saline normal; KLDS6.0930, 2×10^8 CFU *E. durans* KLDS6.0930 labeled with cFDA-SE in 1 mL of sterile normal saline.
